# Autophosphorylation Activity of a Soluble Hexameric Histidine Kinase Correlates with the Shift in Protein Conformational Equilibrium

**DOI:** 10.1016/j.chembiol.2013.09.008

**Published:** 2013-11-21

**Authors:** Marta Wojnowska, Jun Yan, Ganesh N. Sivalingam, Adam Cryar, Jayesh Gor, Konstantinos Thalassinos, Snezana Djordjevic

**Affiliations:** 1 Structural and Molecular Biology, University College London, Gower Street, London WC1E 6BT, UK

## Abstract

In a commonly accepted model, in response to stimuli, bacterial histidine kinases undergo a conformational transition between an active and inactive form. Structural information on histidine kinases is limited. By using ion mobility-mass spectrometry (IM-MS), we demonstrate an exchange between two conformational populations of histidine kinase ExsG that are linked to different levels of kinase activity. ExsG is an atypical signaling protein that incorporates an uncommon histidine kinase catalytic core at the C terminus preceded by an N-terminal “receiver domain” that is normally associated with the response regulator proteins in two-component signal transduction systems. IM-MS analysis and enzymatic assays indicate that phosphorylation of the ExsG receiver domain stabilizes the “compact” form of the protein and inhibits kinase core activity; in contrast, nucleotide binding required for kinase activity is associated with the more open conformation of ExsG.

## Highlights

• Conformational states of a histidine kinase demonstrated with IM-MS
• Phosphorylation of an N-terminal regulatory domain stabilizes compact conformation
• Conformational states are correlated with kinase activity

## Teaser

Wojnowska et al. used ion mobility-mass spectrometry to demonstrate the exchange between two conformational populations of hexameric histidine kinase ExsG. The “open” and “closed/compact” conformations were correlated to the kinase activity and under phosphorylation dependent regulation.

## Introduction

Two-component (TC) signal transduction systems are widely distributed in microorganisms. They can sense diverse stimuli such as nutrients, toxins, light, pH, changes in membrane fluidity, etc., and respond by regulating gene expression or enzymatic activity of a downstream protein or by inducing changes in the direction of cellular movement (Parkinson and Kofoid, 1992, Stock et al., 2000, Gao and Stock, 2009, Krell et al., 2010). A canonical system uses a membrane-embedded histidine kinase (HK) protein with an extracellular or transmembrane sensor (input) region and a cytoplasmic kinase core. Stimulus detection at the sensory domain triggers conformational changes that affect autokinase activity within the C-terminal kinase core; the phosphorylated HK then transfers the phosphoryl group from the phospho-histidine onto the specific aspartate residue within the receiver domain of a cognate response regulator (RR)—the second signaling protein component. Phosphorylation of the receiver domain modulates the activity of the associated effector (output) domain of the response regulator protein that mediates the appropriate response. By using a transmembrane sensor and cytoplasmic regulator protein, microorganisms are able to respond to extracellular changes in the environment, although certain stimuli such as light and diffusible molecules can also be sensed by a subset of soluble HKs found in the cytoplasm.

Prokaryotes with complex lifestyles or diverse metabolic pathways often possess multiple TC signaling systems, all of which use the two basic communication modules—HK catalytic core and the receiver domain (Galperin, 2005, Wuichet et al., 2010, Capra and Laub, 2012). Throughout evolution, these two modules have been extensively duplicated and fused with various input and output domains, providing the versatility of phosphotransfer-based signaling (Wuichet et al., 2010, Capra and Laub, 2012, Alm et al., 2006). Both HKs and RRs constitute families of paralogous genes in which corresponding member proteins exhibit significant sequence identities. Even though the core domains are highly conserved, the signal transduction processes that TC proteins mediate are characterized by high fidelity and lack of cross-reactivity between noncognate pairs. Specificity of protein/protein interactions that is required for directing the appropriate cellular response upon the exposure to the specific stimuli is ensured by coevolution of cognate TC proteins (Capra and Laub, 2012, Laub and Goulian, 2007, Capra et al., 2010).

TC systems are not present in higher eukaryotes and as such, they have been of interest as a possible target for the development of antibacterial agents (Gotoh et al., 2010). More recently, they have become a subject of investigations in the field of synthetic biology. Modular domain organization of TC systems can be exploited by recombinant molecular biology techniques for the design of novel protein domain arrangements with potentially novel in vitro and in vivo activities. In addition, it was demonstrated that signaling outcomes could be redirected by manipulation of the interaction specificities (Capra et al., 2010, Skerker et al., 2008) through site-directed mutagenesis of the residues forming the surfaces at the interface of the HK/RR protein pair. Capacity to generate chimeric proteins with novel functions (Utsumi et al., 1989, Levskaya et al., 2005, Möglich et al., 2009) promotes the use of TC proteins in construction of artificial signaling circuits and biosensors, as well as in designing microbes with unique and programmable properties (Levskaya et al., 2005, Tabor et al., 2009). The successes of these projects depend strongly on the knowledge of structural and functional properties of these proteins including not only description of their domain organization, three-dimensional structure, and kinetic parameters, but also understanding of the dynamics of the conformational transitions associated with signaling and cellular regulation. Although TC systems are found primarily in bacteria, their input/sensory regions contain many signaling domains that are also widely distributed among eukaryotic signaling proteins (i.e., PAS and GAF) and thus TC proteins can be used as model systems for studying general molecular mechanisms that govern intra- and intermolecular signal propagation.

While investigating the domain organization of various TC proteins from the arsenite-oxidizer *Rhizobium* NT-26 (Santini et al., 2000, Andres et al., 2013) we came across a noncanonical TC protein named ExsG. ExsG is encoded within a gene cluster incorporating bacteriophytochrome photoreceptor 1 (BphP1) and two RRs, AgR and ExsF (Karniol and Vierstra, 2003). BphPs are red and far-red light sensors present in several photosynthetic as well as heterotrophic bacteria (Davis et al., 1999, Bhoo et al., 2001, Kumar et al., 2012) and their counterparts in plants and cyanobacteria have been shown to control various light-dependent processes (Smith, 2000). The domain organization of ExsG is uncommon as this protein contains an N-terminal receiver domain linked to the C-terminal histidine kinase domain. Sequence analysis of the histidine kinase of ExsG showed that this protein belongs to the so-called HWE HK family that contains deviations in some of the sequence-motif signatures of the classical HKs (Karniol and Vierstra, 2003, Karniol and Vierstra, 2004). Previously, the autokinase activity of ExsG from *Agrobacterium tumefaciens* has been demonstrated (Karniol and Vierstra, 2004). However, possible regulation of this activity by the N-terminal receiver domain, putative activity of the receiver domain and the potential for ExsG to carry out transphosphorylation to another response regulator has not been investigated. The N-terminal location of the receiver domain within the ExsG also raised the possibility that this protein acts simultaneously as a RR and a HK, potentially relaying the signal between an upstream HK and downstream RR. Furthermore, while only a few three-dimensional structures of the catalytic core of classic HKs are available (Marina et al., 2005, Albanesi et al., 2009, Casino et al., 2009, Yamada et al., 2009, Wang et al., 2013, Diensthuber et al., 2013), and none of them of an active state of the enzyme, no crystal structures of any of the domains of HWE kinases are available. Biochemical and enzymatic properties of this class of kinases have also been poorly characterized. We therefore carried out structural and biochemical characterization of ExsG aiming to expand the knowledge on possible domain arrangements and regulatory mechanisms within the TC systems and potentially enlarge the scope of building blocks available for synthetic and protein engineering efforts.

We present the evidence that ExsG acts as a RR in a branched bacteriophytochrome signaling pathway relaying the signal further as a HK by phosphorylating a downstream cognate RR. By using analytical ultracentrifugation and native mass spectrometry, we show that in contrast to other dimeric histidine kinases the native structure of ExsG is a homohexamer where oligomerization occurs via the HWE HK core. Further, by using ion mobility-mass spectrometry (IM-MS), we were able to demonstrate that the protein exists in equilibrium between the “open/relaxed” and “closed/compact” conformations. Phosphorylation of the ExsG receiver domain appears to stabilize the “closed” state whereby effectively inhibiting autokinase activity. In contrast, binding of an ATP-analog favors the ‘open’ conformation that is consistent with the catalytically active state. This study demonstrates (1) the presence of multiple conformations in the nonstimulated form of the enzyme, and (2) the correlation between activity and global conformation of a histidine kinase as discernible by IM-MS.

## Results and Discussion

### Characterization of the Heterologously Expressed ExsG

**Figure 1:**
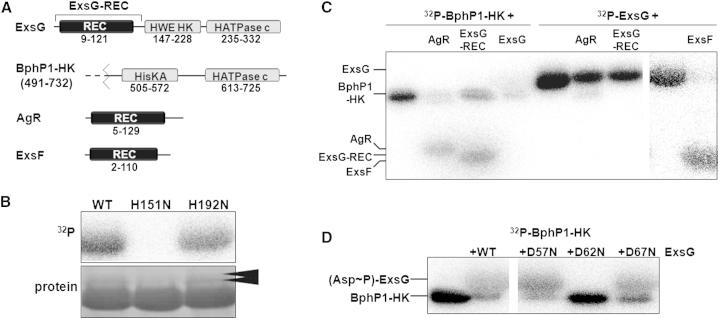
ExsG Phosphorylation Sites and Position within BphP1 Signaling Pathway (A) Schematic diagram of proteins and protein products used in this study. REC domains are shaded black while histidine kinase core domains are shaded gray. HWE HK domain corresponds to dimerization and histidine phosphotransfer domain (DHp) in canonical histidine kinases, and HATPase_c domain corresponds to ATP-binding catalytic (CA) domain. Numbers indicate amino acid residues marking domain boundaries. BphP1 kinase core spanned residues 491–732 of the full-length protein. (B) Autokinase assay of wild-type (WT) ExsG and two histidine mutants. Arrowheads indicate two protein bands migrating in SDS-PAGE with all ExsG variants except the H151N mutant. See also Table S1. (C) Transphosphorylation assays showing phosphotransfer possibilities among BphP1 gene cluster proteins. BphP1-HK and ExsG were allowed to autophosphorylate (30 and 60 min, respectively), added to each putative RR and incubated for 1 hr. Prior to incubation with full-length ExsG and other response regulators, excess of ATP was removed from BphP1-HK autokinase reaction. In the lanes containing RRs in combination with autophosphorylated ExsG, the total amount of radioactivity is reduced due to dilution of the reaction mixtures by the addition of the RR proteins. (D) Transphosphorylation assays (1 hr) verifying phosphotransfer possibility from BphP1-HK onto wild-type ExsG and three aspartate mutants. Excess nucleotide was removed from BphP1-HK autokinase reaction prior to incubations with ExsG variants.

ExsG from *Rhizobium* NT-26 shares 55% sequence identity with a previously identified homolog from a closely related bacterium, *A*. *tumefaciens* (Karniol and Vierstra, 2004). Domain organization assignment carried out with the Simple Modular Architecture Research Tool (SMART; Schultz et al., 1998) annotated an N-terminal response regulator-like receiver domain and a HATPase_c domain in the C-terminal portion of the protein (Figure 1A). HATPase_c domain in SMART corresponds to the ATP-binding region of the catalytic domains present in the GHKL-type ATP-ases including histidine kinases. It should be stressed, however, that similarity to the canonical HATPase_c domain was low (e-value = 9) such that this annotation was only tentative. HATPase_c domain is referred to as CA domain later in the text.

The recombinant full-length ExsG protein was expressed in *Escherichia coli* and purified from the soluble cell extracts. Purified protein exhibited autokinase activity and by means of site-directed mutagenesis, we established that histidine 151 is the site of autophosphorylation (Figure 1B). Intriguingly, SDS PAGE analysis revealed the presence of two additional low intensity protein bands above the main protein band that seemed to be associated with all ExsG variants except for the H151N mutant (Figure 1B). To identify the nature of the associated protein bands that were consistently copurifying, the protein contained in each of the three bands was subjected to tryptic digestion and analyzed using liquid chromatography-tandem mass spectroscopy (LC-MS/MS). MS analysis confirmed that while the main protein band corresponded to ExsG, the two less prominent protein bands were ExsG protein that was covalently modified by the host *E. coli* enzymes. An N-terminal acetylation was detected in the top ExsG protein band and Ser163 phosphorylation was detected in the bottom ExsG band (Table S1 available online). The LC-MS/MS experiment resulted in 50% amino acid sequence coverage; however, one of the peptides not detected in the analysis was the peptide containing His151; we were unable to establish why the H151N mutant does not contain low-scale covalent modifications observed in the wild-type protein.

### ExsG Exhibits Dual Kinase/Regulator Activity

ExsG is encoded within a portion of the NT-26 genome (gene cluster) that contains three other TC-like genes coding for the proteins named BphP1, AgR and ExsF analogously to homologous proteins from *A*. *tumefaciens* (Karniol and Vierstra, 2004). Although the open reading frames coding for these proteins have been identified in *A*. *tumefaciens* the proteins have not been extensively characterized. We hypothesized that these proteins might be functionally linked and involved in a phosphorelay signal transduction. We expressed in *E. coli* and purified the kinase core portion of BphP1 (BphP1-HK, residues 462–703) and the two stand-alone receiver domain proteins AgR and ExsF. In addition, the receiver domain of ExsG was expressed and purified separately (ExsG-REC, residues 1–129) to assess its activity independently of the HWE HK core. While it has been shown previously that *A*. *tumefaciens* AgR receives the phosphoryl group from BphP1 (Karniol and Vierstra, 2003), the potential role of ExsF has not been determined. The results of transphosphorylation assays confirmed not only that AgR is the substrate for BphP1, but also interestingly, the assays showed that the full-length ExsG and the ExsG-REC domain were also efficiently phosphorylated by BphP1-HK (Figure 1C). In these experiments, excess of ATP was removed following autophosphorylation of BphP1-HK and before the addition of RRs (ExsG and AgR) to avoid contribution from the autokinase activity of ExsG, which consequently resulted in relatively slow detected rates of phosphotransfer. We further determined that BphP1 transfers the phosphoryl group on to the ExsG residue Asp62 (Figure 1D) within the conserved response-regulator active site. Transphosphorylation assays indicated that ExsG was capable of transferring the phosphoryl group from the phosphohistidine residue onto ExsF but not AgR or its own receiver domain (Figure 1C). We therefore demonstrate that the four proteins form a TC-like pathway and that the signaling cascade initiated by BphP1 is intrinsically branched, with ExsG potentially acting as a mediator between the photoreceptor and ExsF. Of particular interest to synthetic biology is that in this branched pathway phosphorylation of ExsG is associated with its inhibition and as such it can be considered an “inversion element” (Tabor et al., 2009, Slusarczyk et al., 2012). Within the context of this paper, we are focusing on the regulatory mechanism associated with autokinase activity, and detailed characterization of the signaling pathway including the possible phosphatase activities of BphP1 and ExsG will be described elsewhere.

### Both ExsG and ExsG HWE Core Form a Homohexamer

**Figure 2:**
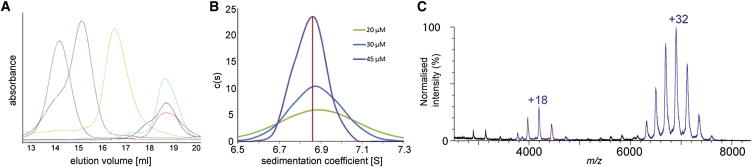
ExsG Homohexamerization via HWE HK Core (A) Overlay of Superose 6 size-exclusion profiles of WT ExsG (brown), ExsG-HK (dark blue), BphP1-HK (green), ExsF (light blue), AgR (red), and ExsG-REC (black). Peak positions were invariant irrespective of loading concentrations. See also Figure S1. (B) Part of the sedimentation coefficient distribution function showing the peak corresponding to ExsG. The distribution was obtained from direct fit to AUC sedimentation velocity data for different loading concentrations of ExsG. The c(s) method was implemented in SEDFIT (Schuck, 2000). (Confirmation of ExsG molecular mass using AUC sedimentation equilibrium experiments is shown in Figure S2.) (C) Native mass spectrum of ExsG showing that ExsG exists predominantly as a hexamer (blue peaks) while small amounts of dimer (purple peaks) are also present.

Classical kinase domains from several transmembrane HK proteins appear to be consistently homodimeric (Gao and Stock, 2009, Marina et al., 2005, Albanesi et al., 2009, Casino et al., 2009, Yamada et al., 2009). BphP1, a cytoplasmic HK, is also likely to form a dimer based on the structure of a homologous protein from *Deinococcus radiodurans* (Li et al., 2010). Because the estimated molecular mass inferred from an ExsG size-exclusion profile appeared to be over 400 kDa, which is approximately 10-fold greater than the monomeric mass (37.6 kDa), we explored the oligomerization state and oligomerization mechanism of ExsG. In addition to wild-type ExsG and ExsG-REC (14.6 kDa), we also expressed and purified the recombinant ExsG kinase core on its own (ExsG-HK, residues 130–336, 23 kDa). Comparison of size-exclusion profiles of the three ExsG protein variants, as well as AgR (17 kDa), ExsF (13.6 kDa), and BphP1-HK (27.2 kDa), indicated the presence of various oligomeric states (Figure 2A). All ExsG protein variants that contain the kinase core appear to form large multimeric complexes with estimated molecular masses of 440 kDa (wild-type ExsG) and 250 kDa (ExsG-HK; Figure 2A), while BphP1-HK exhibited an expected dimeric behavior. In contrast, AgR, ExsF and ExsG-REC domain elution profiles were overlapping, suggesting that these three protein products exhibit similar native molecular mass. Nano-electrospray ionisation (nESI) analysis of ExsF confirmed that this protein exists as a monomer (Figure S1). An additional peak in the elution profiles of both AgR and ExsG-REC domains is indicative of monomer/dimer equilibrium that was previously reported for AgR homologs (Yeh et al., 1997, Im et al., 2002, Benda et al., 2004). Although the receiver domain is not required for the oligomerization of ExsG that is observed for all protein products containing the HWE kinase core, it might be contributing to the stability of the full-length protein and the ExsG hexamer, because the recombinant HWE kinase core was prone to aggregation and precipitation. Classic histidine kinases are characterized by several highly conserved sequence motifs and some of these are only distantly related to (H box) or completely missing in (F box of the ATP lid) the sequence of ExsG HWE kinase core (Karniol and Vierstra, 2004). It is tempting to postulate that distinct structural features of HWE kinase core might be involved in the oligomerization of ExsG.

To determine the mass of the full-length ExsG protein, we used analytical ultracentrifugation, where sedimentation profile monitoring over time provides an estimate of the mass and the shape of the macromolecule. All fits of area under the curve (AUC) sedimentation velocity data converged at a molecular mass of 200–250 kDa with a high frictional coefficient (1.8–1.9) and a sedimentation coefficient of ∼6.9 S, indicative of an elongated hexamer (Figure 2B). AUC sedimentation equilibrium data from several rotor speeds provided further support for the putative hexameric structure (Figure S2). Finally, using nESI-MS, we confirmed that the predominant form of ExsG is a homohexamer with a molecular mass of 226,165.0 ± 36.8 Da (Figure 2C). Given that the receiver domain does not promote oligomerization, we conclude that ExsG hexamerizes via the HWE HK core. This finding constitutes precedence in the field of TC signal transduction, and it raises the question as to whether other HWE HKs also exist as hexamers or if oligomerization is a property associated with soluble histidine kinases (HWE and classic HK).

### The ExsG Hexamer Evolved From Dimers

**Figure 3:**
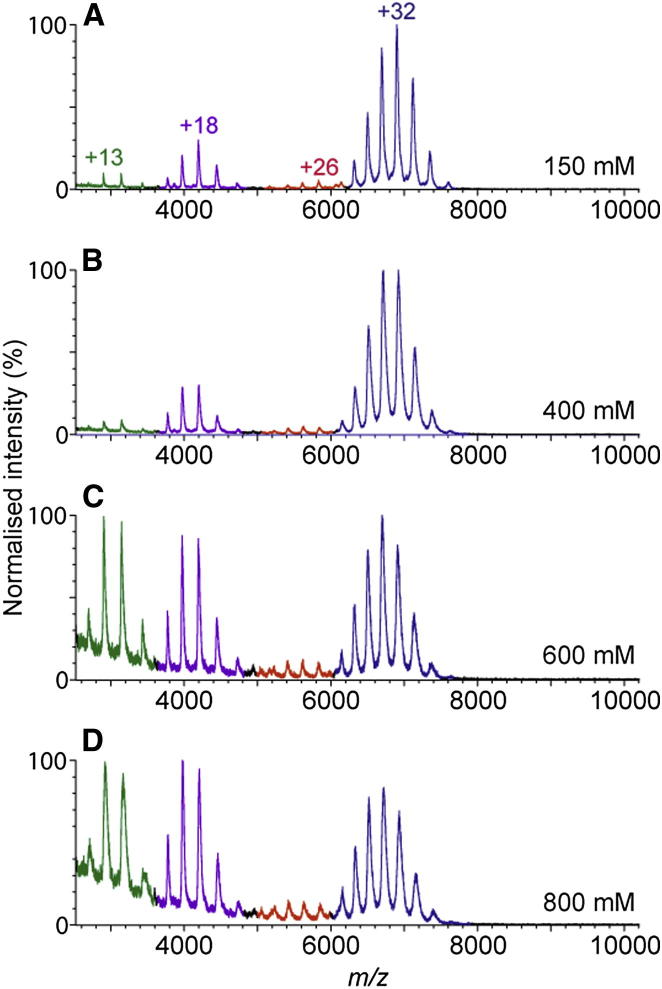
Assembly Pathway of ExsG Monitored by Native Mass Spectrometry Mass spectra showing disruption of the ExsG hexamer with increasing ammonium acetate concentrations. ExsG in (A) 150 mM, (B) 400 mM, (C) 600 mM, and (D) 800 mM ammonium acetate. Charge state series corresponding to hexamer, tetramer, dimer, and monomer, are blue, red, purple, and green, respectively. Deconvolution of oligomeric states was performed using the program Amphitrite (Sivalingam et al., 2013).

To probe the assembly pathway of ExsG, we increased the ionic strength of the ExsG solution to disrupt ionic and polar interactions between subunits and monitored the resulting subcomplexes by means of nESI-MS. This approach has been previously used to perturb subunit interfaces for a large number of protein complexes, where it has been shown that disassembly of a larger molecular complex corresponds to the reverse process of complex assembly under appropriate conditions (Levy et al., 2008, Schmidt et al., 2013, Marsh et al., 2013). Furthermore, it was demonstrated that it is the largest interface in a protein complex that is maintained consistently (80% of cases studied) during disassembly and that the disassembly pathway mimics the evolutionary path of emergence of a molecule (Levy et al., 2008). At low solution ionic strength (Figure 3A), ExsG predominantly exists as a hexamer while small amounts of dimeric form also exist. Upon increasing solution ionic strength (Figures 3C and 3D), the charge state distributions corresponding to the monomer and dimer species increased, as did the distribution of species corresponding to the tetramers. We did not observe any trimer or pentamer species, suggesting that assembly of the hexameric molecule proceeds via association of dimers (trimer of dimers). These data also indicate that the interface in the dimer is larger than in the trimeric interface, suggesting that the hexameric ExsG molecule is evolutionarily linked to a dimeric HK protein that acquired an interaction surface that resulted in formation of a stable hexamer.

### Histidine Kinase Activity of ExsG Is Negatively Regulated by Phosphorylated Receiver Domain

With the capacity of ExsG for His autophosphorylation and downstream phosphotransfer coupled with a potential to receive a phosphoryl group from BphP1 HK, ExsG might play a mediator role, linking stimulus sensed by BphP1 with the response mediated by ExsF. To analyze the role of ExsG receiver domain with respect to the auto-phosphorylation activity of the kinase, we first demonstrated that the presence of the receiver domain is not required for autophosphorylation because a protein variant of ExsG lacking the receiver domain retained kinase activity (Figure S3). The effect of the receiver domain phosphorylation was then examined by using phosphoramidate (PA; a small molecule phosphate donor) and beryllofluoride (BeF_x_) as a phosphomimetic.

**Figure 4:**
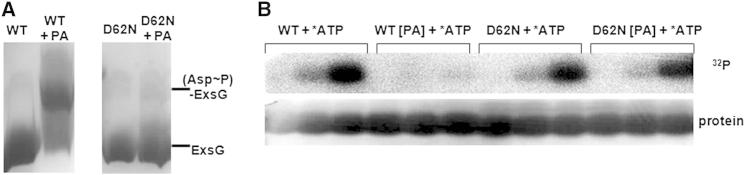
Phosphorylation of ExsG Receiver Domain Inhibits Autokinase Activity (A) Phos-tag/Mn^2+^ gel showing specificity of PA- mediated phosphorylation of ExsG receiver domain. The upshifted band, not observed in the case of ExsG D62N mutant, represents ExsG phosphorylated on Asp62. (B) Autoradiograph of an SDS-PAGE gel demonstrating the effect of ExsG receiver domain phosphorylation on autokinase activity (top). WT and D62N ExsG were incubated with ([PA]) or without PA at RT for 2 hr, purified by spin-column size-exclusion chromatography, and allowed to autophosphorylate upon ATP and Mg^2+^ addition. Samples were taken from each reaction at 5, 15, and 60 min. Coomassie-stained SDS polyacrylamide gel (bottom) confirms that individual lanes contained equal protein levels. Reduced intensity in the first lane of the bottom panel is a staining artifact. See also Figures S3 and S4.

Phos-tag-based phosphoprotein affinity gel electrophoresis was used to show that PA specifically phosphorylates Asp62 (Figure 4A), and we successfully separated ExsG molecules phosphorylated on the aspartate residue within the receiver domain. Under the range of tested conditions, however, those ExsG molecules phosphorylated on the histidine in the kinase core domain migrated together with unphosphorylated protein. Prolonged incubation (2 hr) of wild-type ExsG and D62N mutant with PA resulted in a significant reduction in the extent of WT ExsG autophosphorylation, while PA treatment had no effect on D62N ExsG kinase activity (Figure 4B). D62N mutant, which cannot be phosphorylated on the receiver domain (Figures 1D and 4A), exhibited autokinase activity similar to that of the proteins not subjected to PA (Figure 4B). Incubation of the full-length ExsG with ^32^P-γ ATP in the presence of different concentrations of BeF_x_ also showed a decrease in extent of autophosphorylation with increasing BeF_x_ concentration (Figure S4A). In these experiments, we ensured that BeF_x_ did not interfere with the kinase core or affect the stability of phosphohistidine (Figures S4B–S4D) and that the observed decrease in kinase activity was solely an effect of BeF_x_ interacting with the ExsG receiver domain. In conclusion, treatment of ExsG with either BeF_x_ or PA resulted in decreased levels of ExsG autophosphorylation, demonstrating that phosphorylated receiver domain inhibits HK core activity.

### Phosphorylation of the ExsG Receiver Domain and Nucleotide Binding to CA Domain Induce Opposite Shifts in Conformational Equilibrium of ExsG Hexamer

**Figure 5:**
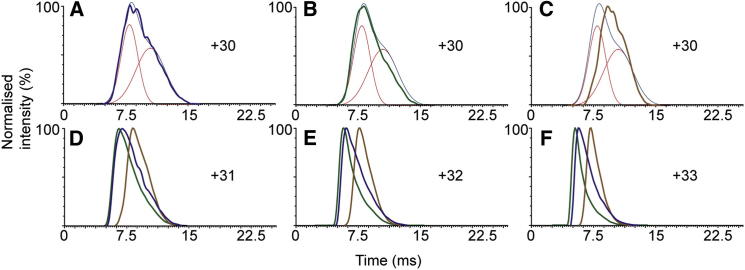
Conformational Changes of ExsG Hexamer upon Phosphorylation by Phosphoramidate and AMP-PNP Binding (A–C) Arrival time distribution of the +30 charge state of the ExsG hexamer with fitted peaks shown in red and the sum of the simulated data shown in light blue (A). ATDs of the +30 charge state of the (B) phosphorylated and (C) AMP-PNP bound ExsG hexamer overlaid on the simulated ATD data of the apo ExsG. (D–F) ATDs of the +31 to +33 charge states of the apo, phosphorylated, and AMP-PNP bound ExsG hexamer. In all plots, arrival time distribution for ExsG alone, phosphorylated ExsG, and ExsG with AMP-PNP bound are blue, green, and brown, respectively. The mass spectra from which the ATDs were extracted are shown in Figure S5.

To probe the conformational plasticity of ExsG and specifically how autophosphorylation and nucleotide binding affect conformation, we studied ExsG alone and upon the treatment with PA or AMP-PNP using ion mobility mass spectrometry. IM-MS measures the time it takes ions, referred to as the arrival time distribution (ATD), to traverse a region filled with inert gas molecules under the influence of a weak electric field. For ions of the same mass and charge, those with a compact conformation will experience fewer collisions with the background gas and, therefore, reach the mass spectrometry detector faster compared to ions of more extended conformations. Many studies have previously shown that under controlled experimental conditions, there is an excellent agreement between IM-MS measurements and those obtained from more established methods used in the field of structural biology (Ruotolo et al., 2005, Scarff et al., 2008). Under all experimental conditions (native, PA, or AMP-PNP treated), ExsG existed primarily as a hexamer (Figure S5). However, our measurements reveal that the ATD, corresponding to the +30 charge state of a native ExsG hexamer (Figure 5A), is fairly broad and asymmetrical. Analysis and fitting of the ExsG profile shows that it can be deconvoluted into two peaks, one at 8 ms and another at about 10.5 ms, consistent with the presence of conformational equilibrium between more compact and more extended forms of the molecule. IM-MS measurements further demonstrate that binding of AMP-PNP and phosphorylation by PA has opposing effects on the conformational equilibrium (Figures 5A–5C). Pretreatment of ExsG with PA favors the population of molecules in a more compact conformation, as the peak in the ATD shifts to the left (Figure 5B), while binding of AMP-PNP results in a decrease in the population of this compact conformation and an increase in the extended form of the molecule, as evidenced by the shift of the peak in the ATD to the right. This behavior was maintained at all charge states that we examined (+30 to +33, Figures 5D–5F). In all experiments, the spectra are normalized to the most intense peak and we are commenting only on the relative populations of the two conformations. Furthermore, it should be acknowledged that the shape of the peaks is relatively broad and that it is possible that each of the two conformations that we refer to either as “open” or “closed” might comprise more complex assembly of energetically closely related conformations sampled by ExsG that cannot be resolved by the current methods. For the simplicity of the argument and in the absence of further data addressing the molecular dynamics of the molecule, we restricted our interpretation and discussion to a dual-state open-active/closed-inactive model. We cannot comment on the detailed nature of the conformational changes and our interpretations are derived from bulk measurements of the hexameric ExsG molecule.

The questions remain regarding the molecular details of the effect that the N-terminal receiver domain of ExsG might have on structure and activity of the histidine kinase. In general, response regulators use different phosphorylation-dependent regulatory strategies (for the review see Gao and Stock, 2010), but effectively all of them involve minor structural perturbation of the REC domain that ultimately affect the molecular surface created by α4-β5-α5 residues of the canonical structural domain. In the case of ExsG, it is possible that phosphorylation of Asp62 results in the formation of a hydrophobic patch on the REC domain which then engages in interaction with the ATP-binding-catalytic (CA) domain of the histidine-kinase core and, in the case of ExsG, inhibits ATP binding and/or hydrolysis. A similar mechanism is observed in regulation of AAA+ activity of the transcriptional activator NtrC (De Carlo et al., 2006). Phosphorylation of the REC domain of NtrC, however, is also linked to the hexamer formation of the AAA+ domain whereas ExsG is intrinsically oligomeric.

### Phosphorylation of the ExsG HK Core Does Not Abolish Receiver Domain Activity

**Figure 6:**
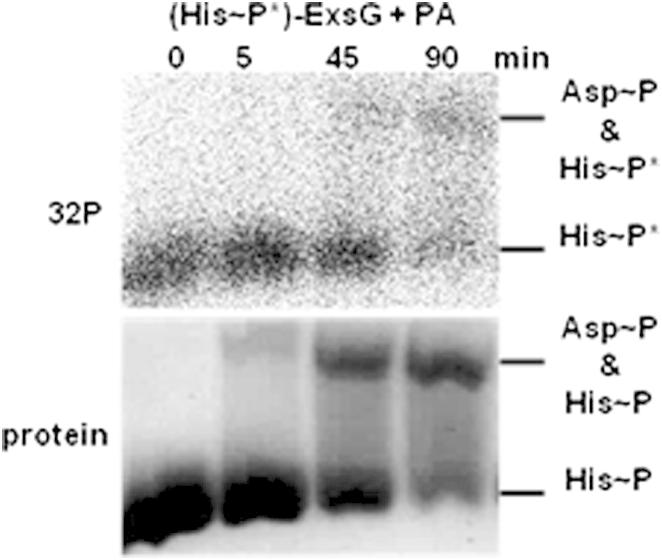
Phosphorylation of ExsG HK Core Does Not Affect the Activity of the Receiver Domain After 1 hr incubation of ExsG with radio-labeled ATP the excess of nucleotide was removed and PA was added. Samples were taken at four time points as indicated and subjected to Phos-tag /Mn^2+^ gel electrophoresis. Autoradiograph (top) shows the gradual emergence of doubly phosphorylated ExsG and the Coomassie-stained gel (bottom) verifies protein levels.

**Figure 7:**
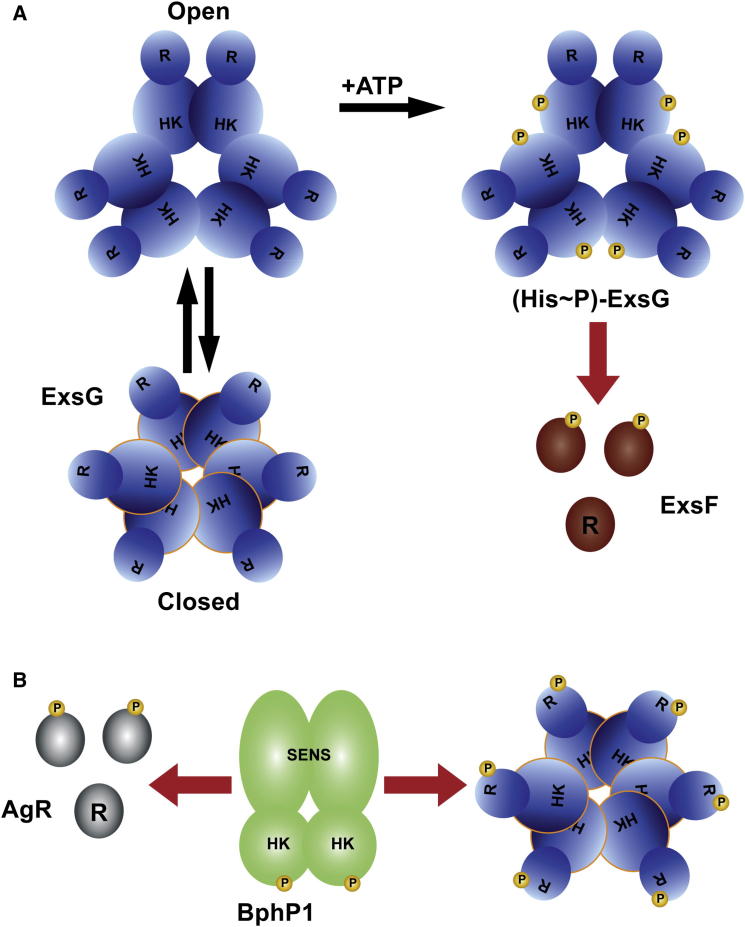
Proposed Model for the Role of ExsG in the BphP1 Signaling Pathway (A) When BphP1 is not phosphorylated, ExsG homohexamers are in equilibrium between “open” and “closed” conformations. The open conformation can bind and hydrolyse ATP, with the phosphoryl group subsequently transferred from ExsG His151 onto the ExsF Asp residue. (B) When BphP1 autophosphorylates, the phosphoryl group is shuttled onto ExsG Asp62, resulting in a stabilization of the closed form of the protein, inhibiting autokinase activity. No phosphotransfer between ExsG and ExsF occurs. We postulate that phosphorylation of AgR by BphP1 stabilizes the dimeric form of this RR in analogy to that observed for Rcp1. R, receiver domain; HK, histidine kinase core; SENS, sensor domain; P, phosphoryl group. Red arrows signify phosphotransfer reaction.

Having established that phosphorylation of the receiver domain results in an inhibition of the kinase domain, by stabilizing the more compact conformational state of the whole protein, we wanted to examine if autophosphorylation on histidine 151 would in turn affect the activity of the receiver domain. The autophosphorylation reaction was carried out first, and after the excess of ATP was removed, PA was added to histidine-phosphorylated ExsG (His-P^∗^)-ExsG. Figure 6 shows that when the proteins were separated by phosphoprotein affinity gel electrophoresis, there was a time-dependent gradual transfer of the radioactive signal from the lower ([His-P]-ExsG) to the upper protein band associated with phospho-Asp containing ExsG. Because only phosphohistidine contained the radiolabel, only those molecules of ExsG that would have phosphoryl groups on both phospho-receiving residues, His151 in the kinase core and Asp62 in the receiver domain, would be visible in the upper band of the autoradiograph. The results indicate that in the conformation adopted by His151-phosphorylated ExsG, REC domain is still accessible to phosphorylation by PA that would be consistent with more relaxed “open” form of the molecule (Figure 7).

### Conclusions

Molecular mechanisms involved in regulation of protein histidine kinase in response to stimuli are still not fully described. Earlier work postulated molecular mechanisms of regulation that included perturbations in conformational equilibrium between the state in which phosphorylatable histidine residue is sequestered and the state where the specific His residue is accessible to the CA domain and the phosphotransfer reaction (Levit et al., 1999, Marina et al., 2005). More recently, information gained from the several structural and biochemical investigations of HKs including tandem DHp and CA domains (Albanesi et al., 2009, Casino et al., 2009, Yamada et al., 2009), and helical HAMP domains, which in many HKs reside between transmembrane helices and DHp domains (Ferris et al., 2012, Mondéjar et al., 2012, Zhu et al., 2013), led to development of new mechanistic models of signal transduction from the sensory domain to the kinase core. In these models, signal propagation by rotary movements, either of the helical linker between the sensory domain and the effector kinase domain (rotary switch), or of the HAMP domain (the gearbox model), induces alterations in interactions between DHp and CA domains resulting in disruption of inhibitory DHp/CA interface and kinase activation (Möglich et al., 2009, Stewart, 2010). In the absence of a crystal structure of an “active” conformation of a histidine kinase, a computational approach was recently described where a structural model of an active state was proposed (Dago et al., 2012). Analysis of the domain contacts coupled with the molecular dynamics simulations suggested that ligand binding to the sensor domain causes localized strain and unwinding of the C-terminal helix within the DHp domain, leading to destabilization of the domain interface and kinase activation. The first crystal structures of HKs comprising sensory, DHp, and CA reported this year (Wang et al., 2013, Diensthuber et al., 2013) strengthened the view that HKs have the capacity and functional requirement to adopt multiple conformations and that the kinase activity is regulated by modulating interactions between DHp and CA domains.

An interesting aspect of ExsG structure is that this molecule exists as a homohexamer, which we believe has evolved from a dimeric HK ancestor. In bacterial chemotaxis signaling systems, transmembrane chemoreceptors form clusters with numerous copies of associated dimeric HKs. It is thought that this arrangement enhances sensitivity of the signaling systems and provides the structural basis for the cooperative behavior of the signaling proteins (Briegel et al., 2009). Electron cryo-tomography images suggest that within the signaling protein clusters, chemotaxis receptors are arranged in hexagonal arrays, most likely formed by trimers of homodimers (Sourjik and Armitage, 2010). Furthermore, a new 3.2 Å resolution crystal structure of ternary complex between the chemotaxis receptor protein interaction region, CheA kinase-regulatory module, and CheW revealed a pseudo 6-fold symmetrical arrangement (Li et al., 2013, Piasta et al., 2013). ExsG adopts a hexameric arrangement and it would be tempting to postulate that oligomerization is associated with the specific signaling properties that this molecule might have, including cooperativity.

## Significance

**Whether it is postulated that there is a single, generic mechanism of histidine kinase activation or that there are several molecular mechanisms evolved to support the signaling function of the diverse pathways, all models rely on the (presumed) existence of the discrete and transient conformational states that HKs adopt. We now show that, at least in the case of ExsG, conformational states could be detected simultaneously and distinguished with IM-MS analysis. Based on our biochemical and activity data in combination with IM-MS, we propose a model of ExsG kinase regulation with the two conformational states described as an open/relaxed/active and a compact/closed/inactive state. In solution, ExsG coexists in two conformations, but the transition from active to an inactive state is favored by the phosphorylation of the N-terminal domain that in this case mimics the sensor domain of the classical HKs. Binding of a nucleotide appears to interfere with the inhibitory conformation of the N-terminal receiver domain, resulting in kinase activation. Recently, crystal structures of nucleotide binding domains of DosS and DosT from *M. tuberculosis* were reported (** **Cho et al., 2013** **). It was shown that in these two HKs that similarly to ExsG lack F box sequence motif and a discernible ATP-lid, kinase activation is regulated by ATP binding in concert with the mechanism of regulation of ExsG that we propose.**

## Experimental Procedures

### Protein Sequence Annotation, Construction of Expression Plasmids, and Protein Purification

Homologs of *A*. *tumefaciens* bacteriophytochrome photoreceptor BphP1 and associated downstream proteins were identified by BLASTP searches against *Rhizobium* NT-26 genome (GenBank FO082822.1). Putative protein domains were assigned by scanning the sequences against the SMART database (Schultz et al., 1998). Sequence alignments were constructed with MUSCLE software (Edgar, 2004) while the secondary structure predictions were performed using the PSIPRED method (Jones, 1999). DNA regions coding for ExsG (CCF21457.1), ExsG-HK (residues 130–336), ExsG-REC (1–129), BphP1-HK (CCF21455.1; residues 462–703), ExsF (CCF21458.1), and AgR (CCF21456.1) were amplified from *Rhizobium* NT-26 genomic DNA using Phusion polymerase (New England Biolabs). PCR products were ligated into NcoI/HindIII cloning sites of a modified pET30a vector in which the enterokinase cleavage site was replaced with the TEV protease recognition site. Site-directed mutagenesis was carried out with QuikChange lightning kit (Agilent Technologies) following recommended manufacturer’s protocols.

Expression of all gene constructs was carried out in *E*. *coli* BL21 (DE3) cells (Agilent Technologies). The cells were grown at 37°C in lysogeny broth supplemented with 50 μg/ml kanamycin until optical density of the cell cultures at 600 nm reached 0.6. Recombinant protein expression was induced with 0.5 mM isopropyl β-D-1-thiogalactopyranoside, and subsequently the cells were kept at 30°C (ExsG and ExsG-HK) or 25°C (all other constructs) for 3–5 hr before being harvested by centrifugation. Cells were lysed by sonication in a buffer containing 50 mM Tris-HCl pH 8.0, 500 mM NaCl, 1 mM β-mercaptoethanol (βME), and the cOmplete EDTA-free protease inhibitors (Roche). BphP1-HK purification buffers additionally contained 5% v/v glycerol. The lysates were centrifuged at >50,000 × *g* and the soluble fractions were incubated for 1 hr with 0.5–2 ml of pre-equilibrated Ni-NTA agarose beads (QIAGEN). Nonspecifically bound proteins were removed from the affinity resin by washing the beads with the sonication buffer containing 20 mM imidazole and His-tagged recombinant proteins were eluted in the presence of 300 mM imidazole. Protein tags were cleaved by in-house purified TEV protease containing uncleavable His-tag that was utilized for the removal of the protease by passing the protein mixture over the pre-equilibrated Ni-NTA beads. The recombinant proteins were further purified by size-exclusion chromatography on Superose 6 (GE Healthcare) column in a buffer containing 50 mM Tris-HCl pH 8.0, 30–500 mM NaCl, and 1 mM βME. All purified protein products were supplemented with 10% v/v glycerol and stored at −20°C.

### Phosphoprotein Affinity Gel Electrophoresis

Phos-tag acrylamide resolving gels contained 7.5 or 10% (w/v) 29:1 acrylamide/N,N′-methylenebisacrylamide, 375 mM Tris-HCl pH 8.0, 0.1% SDS, 100 μM Phos-tag acrylamide, and 200 μM MnCl_2_. Stacking gels contained 4% acrylamide, 125 mM Tris-HCl pH 6.8, and 0.1% SDS. The reactions were stopped with 3× loading buffer (bromophenol blue, 195 mM Tris-HCl pH 6.8, 6% SDS, 30% glycerol, and 2 M βME), and the proteins were separated by electrophoresis at a constant voltage of 160 V in the denaturing running buffer (25 mM Tris-HCl, 192 mM glycine, and 0.1% SDS). To achieve better separation of the protein bands, the electrophoresis was carried out for additional 10–20 min after the dye front reached the bottom of the gel, depending on protein size. Fixing, staining, and destaining were performed according to the Phos-tag acrylamide protocol.

### In Vitro Kinase and Transphosphorylation Assays

Autophosphorylation reactions of the kinases (ExsG wild-type and mutants, ExsG-HK, and BphP1-HK) were performed in a reaction buffer containing 50 mM Tris-HCl pH 8.0, 250 mM NaCl, 1 mM βME, 10 mM MgCl_2,_ 5 mM ATP (Sigma), and 0.4–0.6 μCi/μl [γ-^32^P]-ATP (Perkin Elmer, 6,000 Ci/mmol). Reactions were stopped by adding the appropriate loading buffer, and the phosphorylated proteins were separated using standard 15% SDS Tris-glycine polyacrylamide gels or Phos-tag/Mn^2+^ polyacrylamide gels (7.5% or 10%). Prior to electrophoresis, the excess of ATP in the reaction mixture was removed using Micro Bio-Spin P-6 or P-30 size-exclusion columns (Bio-Rad) where necessary. In transphosphorylation reactions, kinases BphP1 and ExsG were kept at 25 μM and 35 μM, respectively; concentrations of the RRs used were as follows: 60 μM (AgR), 60 μM (ExsG-REC), 60 μM (ExsF), and 50 μM (ExsG). Prior to staining the gels, radiolabeled bands were depicted using a phosphorimaging system (FLA-2000 scanner, Fujifilm) and the signal was quantified in the program MultiGauge (Fujifilm).

### ExsG Receiver Domain Phosphorylation

Potassium phosphoramidate (PA) was synthesized following the standard protocol (Sheridan et al., 2007). To ensure efficient phosphorylation, proteins were incubated in 50 mM Tris-HCl pH 8.0, 250 mM NaCl, and 1 mM βME in the presence of 50 mM MgCl_2_ and 50 mM PA typically for 2 hr unless stated otherwise. The extent of phosphorylation and temporal stability of the phosphorylated receiver domain product was monitored by phosphoprotein affinity gel electrophoresis. Where necessary, prior to loading samples on a gel, the excess of PA was removed using Micro Bio-Spin columns (Bio-Rad).

### Analytical Ultracentrifugation

Sedimentation velocity: ExsG wild-type protein samples in 50 mM Tris-HCl pH 8.0 and 250 mM or 30 mM NaCl were prepared at seven different concentrations (0.2–2 mg/ml) and centrifuged at 40,000 rpm at 20°C for 10 hr in An-Ti50 rotor and Beckman Optima L Series Ultracentrifuge. After the sedimentation velocity run was completed, all samples were analyzed with SDS-PAGE to validate that the protein samples were still intact. Interference data obtained were fitted using the Lamm equation and the continuous c(s) model as implemented in the program SEDFIT (Schuck, 2000).

Sedimentation equilibrium: Wild-type ExsG protein at 6 different concentrations (0.1–1.5 mg/ml) prepared in 50 mM Tris-HCl pH 8.0, and 250 mM NaCl was centrifuged in a Beckman Optima L Series Ultracentrifuge using An-50 Ti rotor at 20°C. Interference and absorbance data were collected at four rotor speeds: 7, 11, 16, and 24 krpm with ten scans per each speed taken every 3 hr. The data were analyzed using SEDFIT and SEDPHAT to obtain the molecular mass of the protein; both single speed and multispeed data files were generated for this purpose.

### Liquid Chromatography-Tandem Mass Spectrometry

Protein bands excised from SDS polyacrylamide gels were digested as described previously (Shevchenko et al., 2006). Peptides were separated using a nanoACQUITY UPLC system coupled to a Synapt HDMS mass spectrometer (Pringle et al., 2007; Waters). The mass spectrometer was operated in a data-independent mode (LC-MS^E^) of acquisition (Silva et al., 2005). Raw data were processed using PLGS v2.5.2.

### Sample Preparation for Native Mass Spectrometry

Purified samples of WT ExsG were concentrated and buffer exchanged to a buffer containing 150 mM ammonium acetate, pH 8, and 0.1 mM dithiothreitol (DTT), using Biospin-6 columns (Bio-Rad). For the nucleotide binding experiment, ExsG protein was incubated with 1 mM AMP-PNP and 2 mM MgCl_2_ at room temperature for 30 min. The incubation mixture was passed through a Biospin-6 column before MS experiments. For the experiments involving phosphoramidate-treated protein, the ExsG protein was incubated with 50 mM MgCl_2_ and 50 mM potassium phosphoramidate for 3 hr, and passed through two Biospin-6 columns before MS experiments. For the assembly pathway experiments, the ExsG protein was buffer-exchanged to buffers containing, 400 mM, 600 mM, and 800 mM ammonium acetate, pH 8, respectively, and 0.1 mM DTT using Biospin-6 columns. Spraying concentration of the protein in all experiments was kept at 30–40 μM (based on monomer).

### Ion Mobility Spectrometry-Mass Spectrometry

Mass spectrometry experiments were carried out on a first-generation Synapt HDMS (Waters, Manchester, UK) Quadrupole-TOF, traveling wave ion mobility mass spectrometer (Pringle et al., 2007). With use of gold-coated capillaries prepared in house 2.5 μl aliquots of the protein sample were introduced to the mass spectrometer by means of nESI ionization. Typical instrumental parameters were: source pressure 5.5 mbar; capillary voltage, 1.1 kV; cone voltage, 30 V; trap energy, 8 V; and transfer energy, 6 V. The IMS cell pressure was 5.20 × 10^−1^ mbar, IMS wave velocity 250 m/s, IMS wave height 9, and trap pressure 4.07 × 10^−2^ mbar. ATD peak fitting was performed using the program Fityk 0.9.8 (Wojdyr, 2010). Peaks were modeled as Gaussian and fitted to the ATDs using the Levenberg–Marquardt algorithm.

## Supplemental Information

Document S1. Figures S1–S5

Table S1. Analysis of ExsG Protein
