# Supplementary material for: Autophosphorylation Activity of a Soluble Hexameric Histidine Kinase Correlates with the Shift in Protein Conformational Equilibrium
Source: Chem Biol. 2013 Nov 21;20(11):1411–20. doi: 10.1016/j.chembiol.2013.09.008 (PMC3899027; doi:10.1016/j.chembiol.2013.09.008)
Supplement: Document S1. Figures S1–S5 [file mmc1.pdf]

Chemistry & Biology, Volume *20*

Supplemental Information

Autophosphorylation Activity of a Soluble Hexameric Histidine Kinase Correlates with the Shift in Protein Conformational Equilibrium

Marta Wojnowska, Jun Yan, Ganesh N. Sivalingam, Adam Cryar, Jayesh Gor, Konstantinos Thalassinos, and Snezana Djordjevic

Table S1 related to Figure 1B. LC-MS/MS analysis of the two SDS-PAGE protein bands associated with the main recombinant ExsG protein band following purification. The protein contained in each of the bands was subjected to tryptic digestion and analyzed using LC-MS/MS. The table shows molecular weights of the MS peaks and the sequences of the identified peptides.

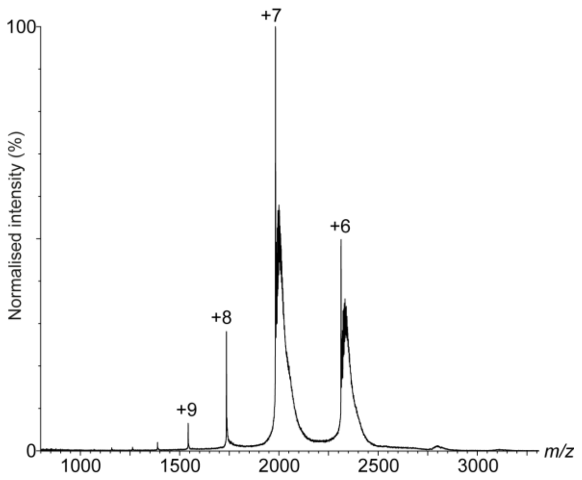

Figure S1 related to Figure 2. ExsF exists as a monomer. Native mass spectrum of ExsF showing the protein exists as a monomer.

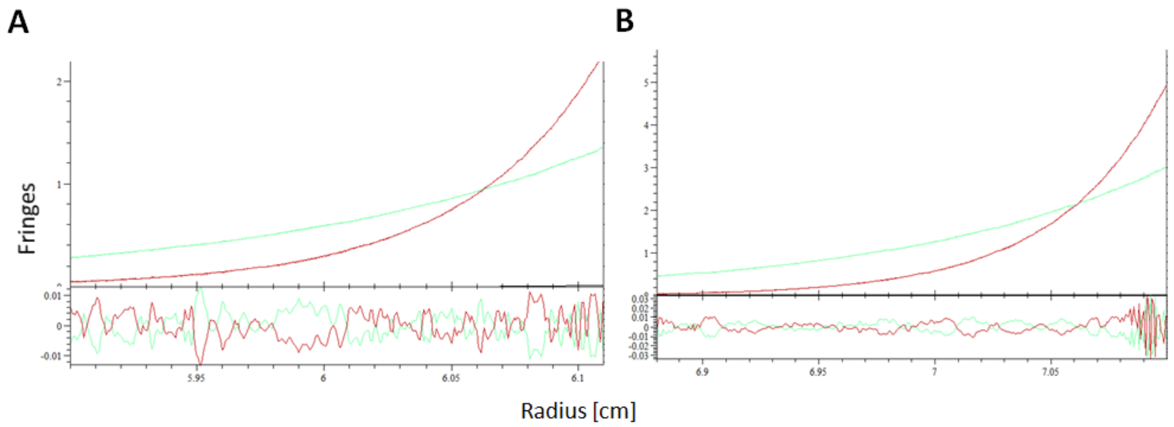

Figure S2 related to Figure 2. Confirmation of ExsG molecular mass using AUC sedimentation equilibrium. Examples of fits to SE multi-speed interference data using

“single species of an interacting system” model with a fixed mass parameter (226 kDa) implemented in SEDPHAT (Vistica J, et al. (2004) Sedimentation equilibrium analysis of protein interactions with global implicit mass conservation constraints and systematic noise decomposition. *Analytical Biochemistry* 326:234-256). The RMSD values were 0.0047 (A, ExsG concentration 5 μM) and 0.008 (B, ExsG concentration 8 μM) for the individual fits. Residual plots are shown below the each graph. Green – equilibrium concentration gradient at 7 krpm, brown – at 11 krpm.

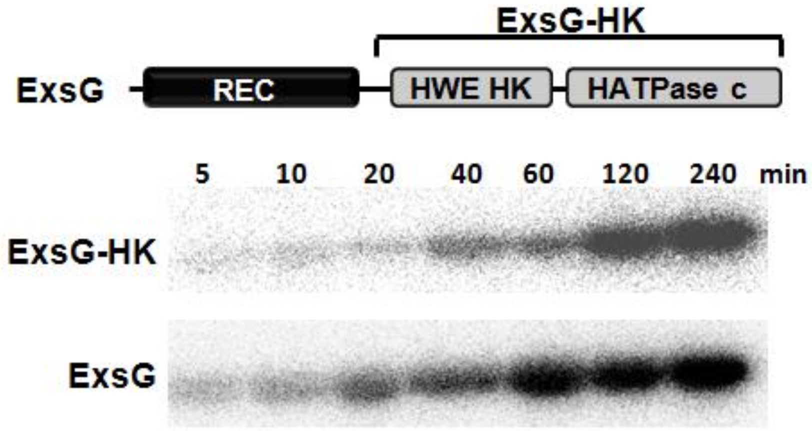

Figure S3 related to Figure 4. Time-course autokinase assays of full-length ExsG and ExsG-HK. Both proteins were present at a concentration of 30 μM.

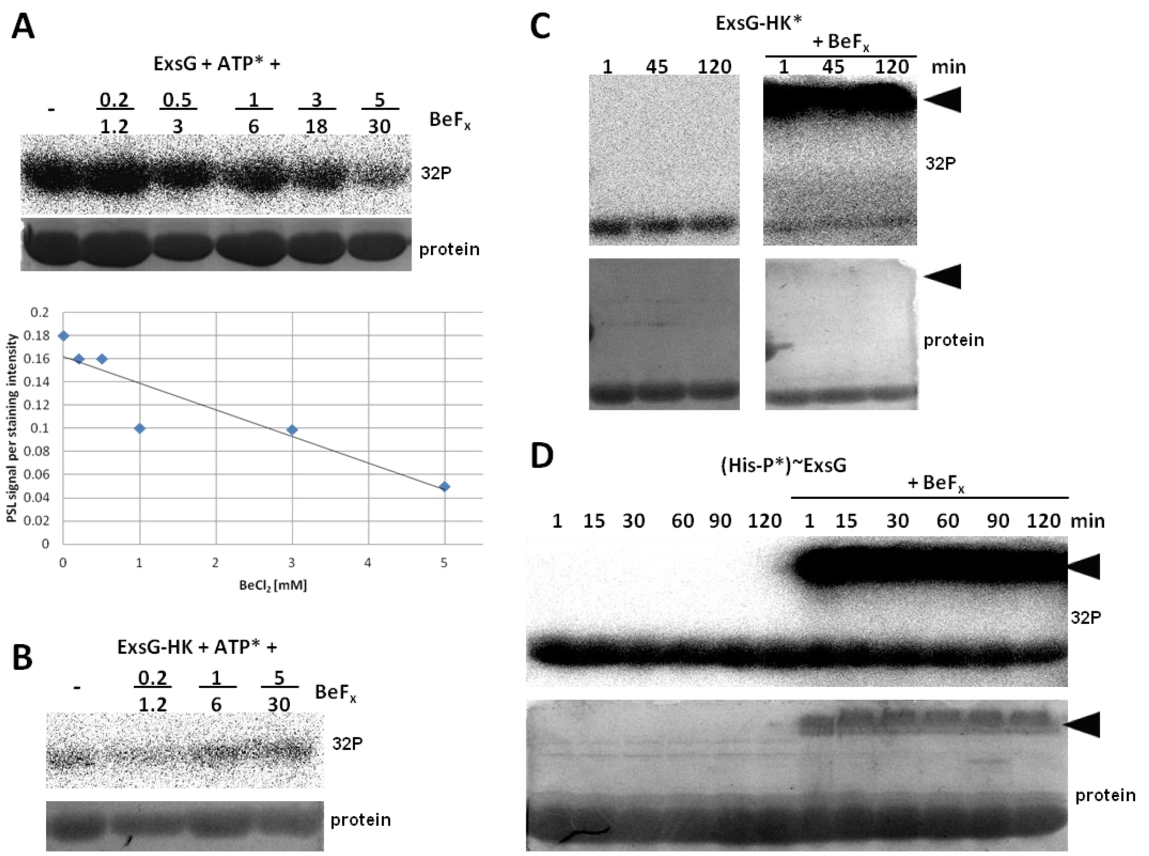

Figure S4 related to Figure 4. Effect of beryllofluoride on ExsG autokinase activity. (A) The plot (bottom) reflects the dependence of ExsG HK activity on beryllofluoride concentration as seen on the autoradiograph (top). Ratios indicate the concentration of beryllium chloride (top value) and sodium fluoride (bottom value). PSL signal was standardised per staining intensity of each band and the relative PSL signal was plotted against the concentration of beryllium chloride. (B) Autoradiograph showing ExsG-HK autokinase activity in the presence of increasing beryllofluoride concentrations. (C) and (D) Autoradiographs demonstrating the stability of ExsG-HK (C) and ExsG wild-type (D) phosphohistidine in the presence and absence of 5 mM BeCl2 and 30 mM NaF. Black arrows indicate highly radioactive protein aggregates observed when beryllofluoride is present at a high concentration and is not removed prior to loading on SDS polyacrylamide gel.

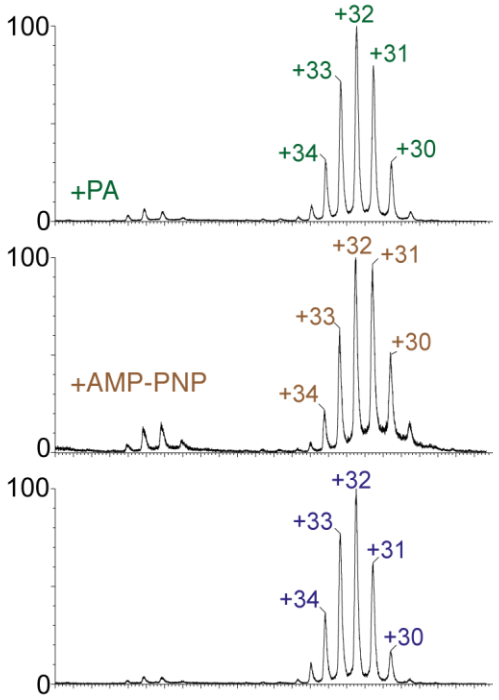

Figure S5 related to Figure 5. Mass spectra of ExsG and ExsG bound to AMP-PNP and PA. The spectra were obtained in ion mobility mode and were used to extract the arrival time distributions shown in Figure 5. Mass spectral peaks used to extract arrival times are +31, +32 and +33.
